# Long noncoding RNA NEAT1 regulates the development of osteosarcoma through sponging miR‐34a‐5p to mediate HOXA13 expression as a competitive endogenous RNA

**DOI:** 10.1002/mgg3.673

**Published:** 2019-05-01

**Authors:** Shaolin Ji, Shunsheng Wang, Xiaodan Zhao, Li Lv

**Affiliations:** ^1^ Hand Foot Surgery Yidu Central Hospital of Weifang City Qingzhou China; ^2^ Anorectal Surgery Yidu Central Hospital of Weifang City Qingzhou China; ^3^ Thoracic Surgery The First Hospital of Xingtai City Xingtai China; ^4^ Hand Surgery The Third Hospital of Hebei Medical University Shijiazhuang China

**Keywords:** apoptosis, long noncoding RNA, osteosarcoma, proliferation

## Abstract

**Background:**

Long noncoding RNA (lncRNA) exerts a potential regulatory role in tumorigenesis. LncRNA *NEAT1* expression remains high in osteosarcoma tissues. However, its biological mechanism in osteosarcoma remains unknown.

**Methods:**

In this study, *NEAT1* expression in osteosarcoma cells was detected by qRT‐PCR. Proliferative and apoptosis potentials of osteosarcoma cells were determined by CCK‐8 assay and Flow Cytometry, respectively. We identified the potential target of *NEAT1* through bioinformatics and dual‐luciferase reporter gene assay. Furthermore, their interaction and functions in regulating the development of osteosarcoma were clarified by Western blot and RIP assay.

**Results:**

Our results demonstrated a high expression of *NEAT1* in osteosarcoma tissues and cells. Overexpression of *NEAT1* markedly accelerated proliferative and reduced apoptosis potentials of osteosarcoma cells. Besides, *NEAT1* could positively regulate the expression of *HOXA13* by competing with miR‐34a‐5p.

**Conclusion:**

These results indicated that *NEAT1* participated in the development of osteosarcoma as a ceRNA to competitively bind to miR‐34a‐5p and thus mediate *HOXA13* expression.

## 1 INTRODUCTION

Osteosarcoma is common in children and teenagers (Anderson, 2016), whose incidence is second only to lymphoma among malignant tumors in teenagers. Osteosarcoma accounts for about 3%–4% of all pediatric tumors and 30% of malignant bone tumors (Moore & Luu, 2014; Wycislo & Fan, 2015), and it often occurs in the epiphysis and other sites near the knee, proximal tibia, and distal femur (Isakoff, Bielack, Meltzer, & Gorlick, 2015). Many patients with osteosarcoma have varying degrees of lung metastasis when they are first diagnosed (Zhou et al., 2014). If lung metastasis occurs, the 5‐year‐survival rate of these patients will be less than 20% (Friebele, Peck, Pan, Abdel‐Rasoul, & Mayerson, 2015). Currently, the focus of research on osteosarcoma has been extensively and gradually extended to genetic, molecular, and protein levels. For example, Li, Dou, Liu, & He (2017) published a paper *Application of Long Noncoding RNAs in Osteosarcoma: Biomarkers and Therapeutic Targets*. Zhang et al. (2016) found that the level of long noncoding RNA (lncRNA) *MALAT1* in osteosarcoma tissues was higher than that in paired adjacent normal tissues, and it affects the invasion and metastasis abilities of osteosarcoma cells. To further explore the pathogenesis of osteosarcoma, the mechanisms of the occurrence, progression and apoptosis of osteosarcoma were elucidated at the epigenetic level. Our results are of great significance to improve the diagnostic and therapeutic approaches of osteosarcoma.

LncRNAs are noncoding RNAs with more than 200 nucleotides that are capable of regulating gene expressions (Lorenzen & Thum, 2016; Sun, Yang, Xu, & Guo, 2017). They have been widely concerned in recent years due to their complex biological functions. It is reported that certain lncRNAs exert their crucial effects on proliferation, apoptosis, invasion, and infiltration of many types of tumor cells (Chen, Xu, & Zhang, 2017; Mao et al., 2017; Min et al., 2016; Wang et al., 2017). LncRNA Nuclear Enriched Abundant Transcript 1 (*NEAT1*, NCBI Gene ID: 283131) is an lncRNA with diverse functions in tumorigenesis (Dong et al., 2018; Ghafouri‐Fard & Taheri, 2019; Yang et al., 2017). Previous studies have indicated that *NEAT1* participates in the pathogenesis of various diseases, such as nervous system diseases, cardiovascular diseases, and various tumors (Ahmed et al., 2018; Fujimoto et al., 2016; Sunwoo et al., 2017). The promotive role of *NEAT1* has been identified in hepatocellular carcinoma (Fang et al., 2017) and ovarian cancer (An, Lv, & Zhang, 2017).

In this study, we examined *NEAT1* in osteosarcoma tissues and adjacent noncancerous tissues. Our results verified that *NEAT1* was highly expressed in osteosarcoma tissues. The biological role of *NEAT1* in the pathological process of osteosarcoma has been pointed out (Li et al., 2018). However, the specific mechanism of *NEAT1* involvement in osteosarcoma development still needs to be explored.

## 2 MATERIALS AND METHODS

### 2.1 Ethical compliance

The research was approved by the Ethics Committee of The Third Hospital of Hebei Medical University.

### 2.2 Specimen collection and processing

In this study, 72 pairs of osteosarcoma tissues and matched adjacent normal tissues were collected through surgery and osteosarcoma was confirmed by postoperative pathological examination. Immediately after the fresh tissues from the lesion and the normal tissue adjacent to the tumor were removed by professional physicians using special forceps, these tissues were washed with DEPC, put into the refrigerated tube, and placed in the labeled liquid nitrogen tank for freezing. Subsequently, the clinical data were collected in the department of pathology. Tissue sample collection was approved by the patients and the Ethics Committee. Among these patients, there were 26 females and 46 males aged between 12 and 31, with an average age of 18.3 ± 5.7.

### 2.3 Cell culture and transfection

Human normal osteoblast cell line hFOB1.19 and osteosarcoma cell lines Saos2, MG63, U2OS, SJSA1, and HOS were purchased from ATCC (Manassas VA). Cells were cultured in DMEM containing 10% FBS (Beyotime, Nantong, China), 100 μg/ml streptomycin, and 100 IU/ml penicillin (Invitrogen, USA), and maintained under 5% CO_2_ at 37°C. *NEAT1* overexpression plasmid, *NEAT1* siRNA, miR‐34a‐5p mimics and miR‐34a‐5p inhibitor were all constructed by GenePharma (Shanghai, China). Transfection was performed using Lipofectamine 2000 (Invitrogen, CA).

### 2.4 RNA extraction and qRT‐PCR

Total RNA was isolated using the TRIzol Reagent (Invitrogen, USA). Using the Reverse Transcription Kit (Takara, Tokyo, Japan), cDNA was obtained from reverse transcription of RNA. Subsequently, an ABI 7900 system (Applied Biosystems, CA) and SYBR Green PCR kit (TaKaRa Biotechnology, Dalian, China) were adopted for qRT‐PCR. With GAPDH as an internal control, the lncRNA expression level was normalized, and 2^−ΔCt^ method was adopted to assess the fold change in lncRNA expression. Primer sequences are displayed below.

*NEAT1*: F 5′ TCTGTGTGTCAAAGCAAGGC 3′,

R 5′ AGATGCCACTGAATCACCCA 3′,

*HOXA13*: F: 5′ CTGCCCTATGGCTACTTCGG 3′;

R: 5′ CCGGCGGTATCCATGTACT 3′.

*GAPDH*: F: 5′ CCGGGAAACTGTGGCGTGATGG 3′,

R: 5′ AGGTGGAGGAGTGGGTGTCGCTGTT 3′,

### 2.5 Cell proliferation assays

Cells were cultured in 96‐well plates and incubated with CCK‐8 reagent (Beyotime, Nantong, China) for 1 hr. The absorbance at 450 nm was recorded using a TECAN infinite M200 Multimode microplate reader (Tecan, Mechelen, Belgium).

### 2.6 Cell apoptosis assays

Cells were dyed with Annexin V‐FITC and propidium iodide (PI), and apoptosis was determined by flow cytometry (BD Biosciences, Franklin Lakes, NJ). Apoptosis was assayed through dual‐staining with annexin V‐FITC and propidium iodide (PI; BD Biosciences, Franklin Lakes, NJ). In brief, transfected cells for 48 hr were incubated with Annexin V‐FITC and PI in dark, and subjected to analysis by flow cytometry (BD Biosciences, Franklin Lakes, NJ).

### 2.7 Subcellular distribution

RNAs in cytoplasm and nucleus were extracted with the PARIS Kit (Life Technologies, USA). Total RNA in each fraction was quantified by qRT‐RCR. GAPDH and U6 were utilized as cytoplasm and nucleus internal references, respectively.

### 2.8 Dual‐luciferase reporter gene assay

Wild‐type plasmids *NEAT1*‐WT and *HOXA13*‐WT, as well as mutant‐type plasmids *NEAT1*‐MUT and *HOXA13*‐MUT were constructed. HOS and Saos2 cells seeded into 24‐well plates were co‐transfected with 50 nmol/L miR‐34a‐5p mimics or a negative control and wild‐type or mutant‐type plasmid using Lipofectamine 2000. 5 ng of pRL‐SV40 was added per 80 ng of plasmid. Dual‐luciferase reporter assay kit (Promega, Madison, WI) was used for determining luciferase intensity on a microplate reader.

### 2.9 RNA‐binding protein immunoprecipitation (RIP)

The Magna Nuclear RIP^™^ (Native) Nuclear RIP Kit (Millipore, Bedford, MA) was used for RIP assay. Cells were lysed in complete RIPA buffer containing protease inhibitor cocktail and an RNase inhibitor. Cell extract was incubated with RIP buffer containing magnetic beads conjugated to human anti‐AGO2 antibody (Millipore) or IgG control. Immunoprecipitated RNA was obtained from protein digestion. Finally, the purified RNA was quantified by qRT‐PCR. Anti‐*NEAT1* used for RIP assay was purchased from Abcam (Cambridge, MA).

### 2.10 Western blot

Protein samples were extracted and quantified by BCA, separated by SDS‐PAGE gel electrophoresis, and blocked with 5% skim milk. Membranes were then incubated with the primary antibodies (rabbit anti‐human IgG antibodies against *HOXA13* and GAPDH) and corresponding secondary antibodies. Bands were exposed, and the color was developed by chemiluminescence.

### 2.11 Statistical processing

SPSS 20.0 software and GraphPad Prism 6.0 were utilized for statistical analysis. Quantitative data were represented as mean ± *SD*. Measurement data were analyzed by the *t* test, whereas data not conformed to the normal distribution were subjected to nonparametric test. The value of *p* < 0.05 indicated statistical significance.

## 3 RESULTS

### 3.1 *NEAT1* expression and function in osteosarcoma

**Figure 1:**
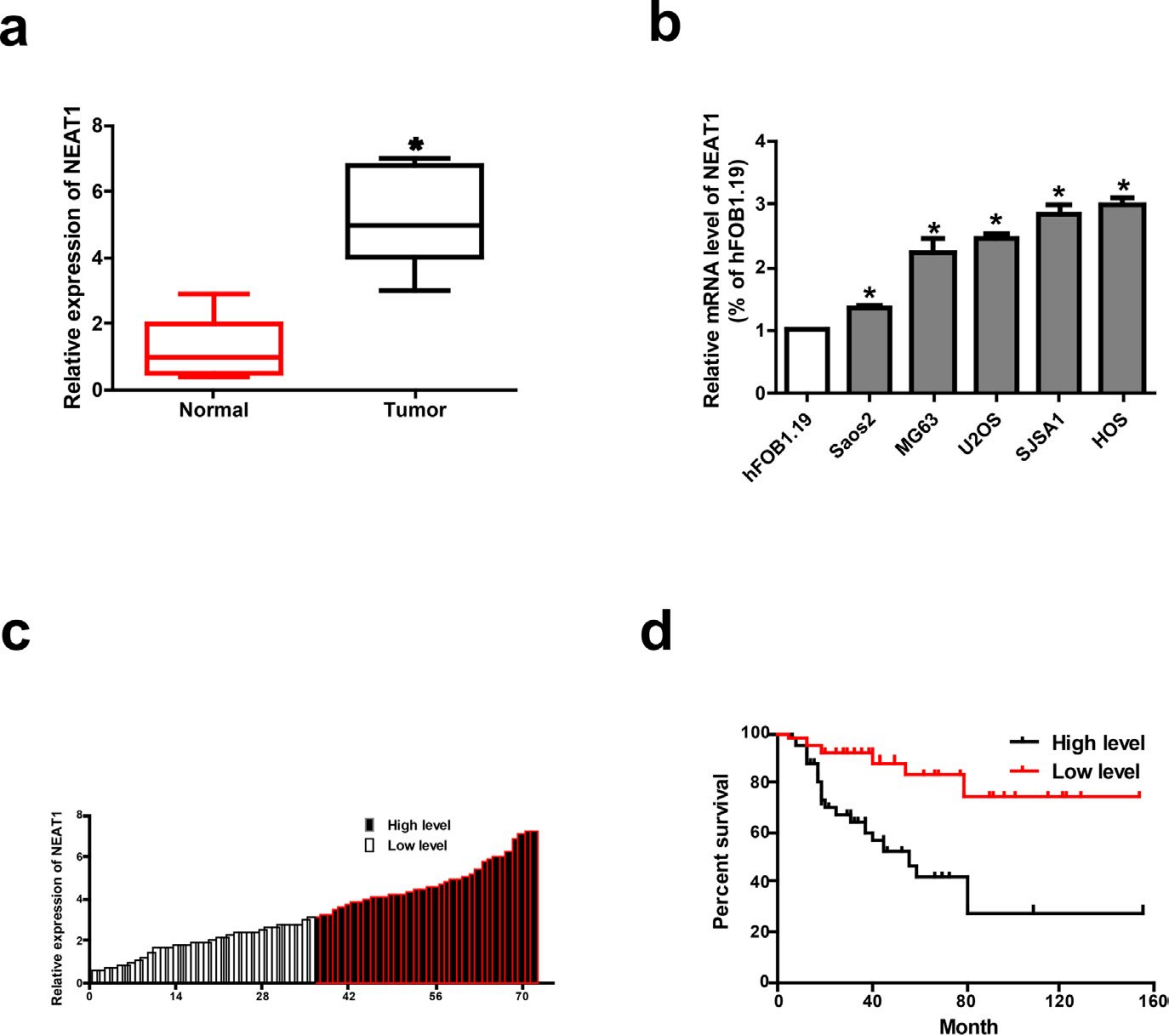
High expression of *NEAT1* in osteosarcoma. (a) *NEAT1* is highly expressed in osteosarcoma tissues. (b) *NEAT1* expression in osteosarcoma cell lines (Saos2, MG63, U2OS, SJSA1, HOS) and human normal osteoblast cell line hFOB1.19 detected by qRT‐PCR. (c) The heights of the columns in the chart represented the log2‐transformed fold changes (osteosarcoma tissues/normal tissues) in *NEAT1* expression in 72 patients with osteosarcoma. (d) Correlation between *NEAT1* expression and overall survival of patients with osteosarcoma detected by Kaplan–Meier analysis. Data are presented as mean ± *SD*. *p < 0.05

*NEAT1* expression in osteosarcoma tissues and paired paracancerous tissues was detected by qRT‐PCR. The results showed that *NEAT1* was highly expressed in osteosarcoma tissues (Figure 1a). We then examined the relative expression of *NEAT1* in osteosarcoma cells (Saos2,MG63, U2OS, SJSA1, and HOS) and human normal osteoblast cells hFOB1.19 by qRT‐PCR. The above results indicate that *NEAT1* is highly expressed in osteosarcoma cells, which is consistent with the results found by Wang, Yu, Fan, & Luo (2017). In particular, HOS cells expressed the highest level, while Saos2 cells expressed the lowest level of *NEAT1* among the selected osteosarcoma cell lines, which were chosen for the subsequent experiments (Figure 1b). Additionally, the prognostic value of *NEAT1* expression was determined for overall survival in osteosarcoma patients by the Kaplan–Meier analysis, suggesting that high expression level of *NEAT1* is significantly correlated with shorter overall survival (Figure 1c and d).

### 3.2 *NEAT1* facilitates cell proliferation and inhibits apoptosis signaling

**Figure 2:**
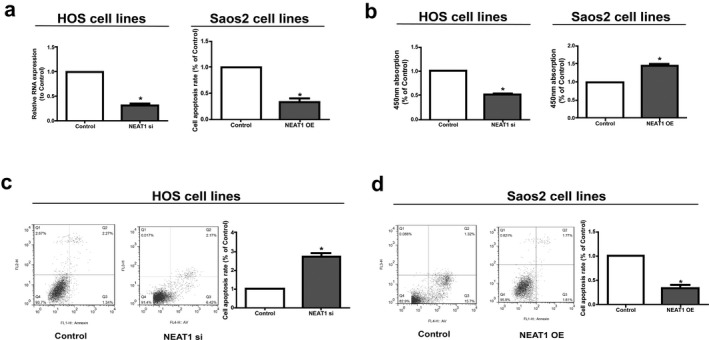
Regulatory effect of *NEAT1* on proliferation and apoptosis of osteosarcoma cells. (a) After knock‐down of *NEAT1* in HOS cell line and overexpression of *NEAT1* in Saos2 cell line, we calculate their transfection efficiency. (b) CCK‐8 assay showed proliferation of HOS transfected with *NEAT1* siRNA and Saos2 cells transfected with *NEAT1* overexpression vector. (c and d) Cell apoptosis detected by BD Biosciences FACS Calibur Flow Cytometry. Data are presented as mean ± *SD*. *p < 0.05

After knock‐down of *NEAT1* in HOS cell line and overexpression of *NEAT1* in Saos2 cell line, we calculate their transfection efficiency (Figure 2a). Then, CCK‐8 assay indicated that *NEAT1* downregulation markedly decreased the proliferative ability of osteosarcoma cells. *NEAT1* overexpression accelerated the proliferative rate of osteosarcoma cells (Figure 2b). In addition, cell apoptosis experiment showed that overexpression of *NEAT1* reduced the apoptosis capacity of osteosarcoma cells, and *NEAT1* downregulation induced the apoptosis capacity of osteosarcoma cells (Figure 2c and d). Taken together, these results indicate that *NEAT1* may exert regulatory effects on apoptosis and proliferative potentials of osteosarcoma cells.

### 3.3 Subcellular distribution of *NEAT1*

**Figure 3:**
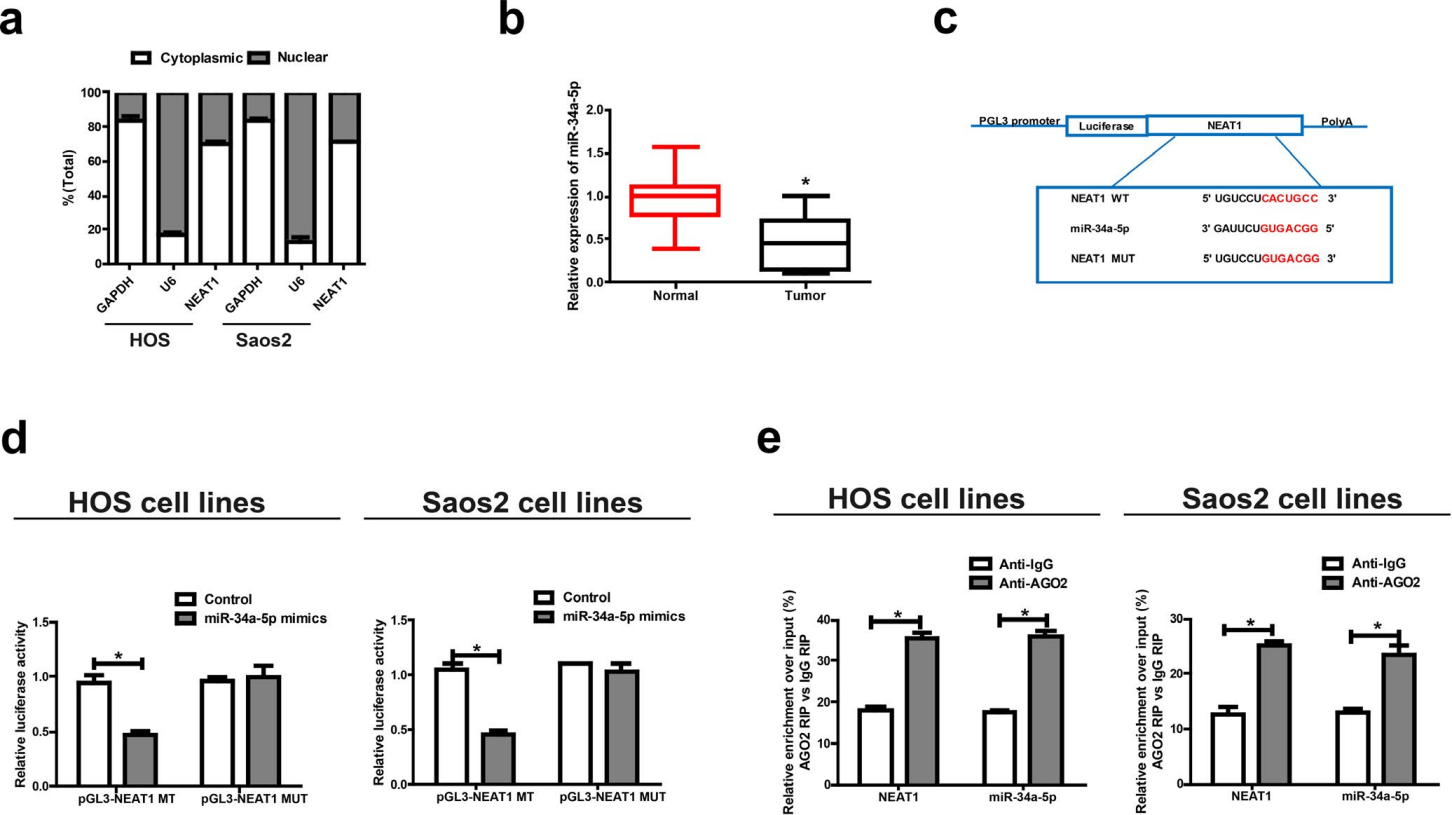
*NEAT1* directly interacts with miR‐34a‐5p. (a) Cytoplasmic and nuclear levels of *NEAT1* in HOS and Saos2 cells analyzed by qRT‐PCR. (b) MiR‐34a‐5p expression in osteosarcoma tissues. (c) Bioinformatics evidence of binding of miR‐34a‐5p onto 3′‐UTR of *NEAT1*. (d) Dual‐luciferase reporter gene assay in HOS and Saos2 cells after transfection with negative control or miR‐34a‐5p mimics, renilla luciferase vector pRL‐SV40 and the reporter constructs. (e) RIP experiments for the amount of *NEAT1* and miR‐34a‐5p in HOS and Saos2 cells. Data are presented as mean ± *SD*. *p < 0.05

Subcellular distribution of lncRNA determines its biological function. To confirm the cellular localization of *NEAT1*, we isolated osteosarcoma cells into cytoplasmic and nuclear fractions, with GAPDH and U6 as controls, respectively. QRT‐PCR results showed that 69.5% and 71.05% of *NEAT1* were distributed in the cytoplasmic fraction of HOS and Saos2 cells, respectively (Figure 3a). We may conclude that *NEAT1* participates in the development of osteosarcoma through post‐transcriptional regulation.

### 3.4 *NEAT1* is targeted by miR‐34a‐5p

Given that *NEAT1* was primarily located in the cytoplasmic fraction, we hypothesized that *NEAT1* may act as a ceRNA in the development of osteosarcoma. QRT‐PCR data revealed that miR‐34a‐5p expression was lower in osteosarcoma tissues, which was contrary to the expression trend of *NEAT1* (Figure 3b). Through RegRNA, Starbase prediction, we found that sequences in miR‐34a‐5p that were highly matched to *NEAT1* 3′UTR. Based on these binding sequences, we constructed pGL3‐*NEAT1*‐WT and pGL3‐*NEAT1*‐MUT (Figure 3c). Luciferase activity was obviously downregulated in HOS and Saos2 cells co‐transfected with *NEAT1* WT and miR‐34a‐5p mimics, while it did not change after transfection with *NEAT1* MUT (Figure 3d). RIP analysis was carried out to elucidate whether *NEAT1* was involved in RNA‐containing ribonucleoprotein complex. QRT‐PCR results showed that *NEAT1* was enriched in anti‐AGO2 antibody compared with in controls. Similar results were yielded in miR‐34a‐5p (Figure 3e). The above results imply that miR‐34a‐5p can bind to *NEAT1* in vitro.

### 3.5 *NEAT1* regulates *HOXA13*, the target gene of miR‐34a‐5p

**Figure 4:**
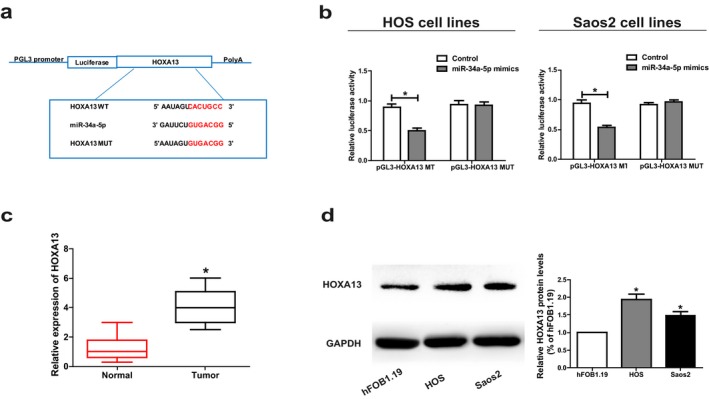
*HOXA13* is the direct target of miR‐34a‐5p. (a) The putative miRNA‐binding sites in the *HOXA13* sequence. (b) Direct target sites confirmed by dual‐luciferase reporter gene assay. (c) *HOXA13* expression in osteosarcoma tissues. (d) Protein level of *HOXA13* in hFOB1.19, HOS and Saos2 cell lines detected by Western Blot. Data are presented as mean ± *SD*. *p < 0.05

To investigate the potential role of miR‐34a‐5p in the development of osteosarcoma, we screened out the target genes of miR‐34a‐5p by bioinformatics prediction (TargetScan, Starbase, RegRNA). Finally, *HOXA13* was selected for further analyses. After construction of luciferase plasmids pGL3‐ *HOXA13*‐WT and pGL3‐ *HOXA13*‐MUT, they were cotransfected with miR‐34a‐5p mimics or NC in HOS and Saos2 cells, respectively (Figure 4a). Luciferase activity of the WT reporter was inhibited, while MUT reporter group did not change (Figure 4b). The above results indicate that *HOXA13* is a potential target gene of miR‐34a‐5p. Subsequently, *HOXA13* expression in osteosarcoma tissues and paired paracancerous tissues was determined by qRT‐PCR. The mRNA levels of *HOXA13* were remarkably elevated in osteosarcoma tissues compared to paracancerous tissues (Figure 4c). Western blot analysis revealed the same result at the protein level (Figure 4d).

**Figure 5:**
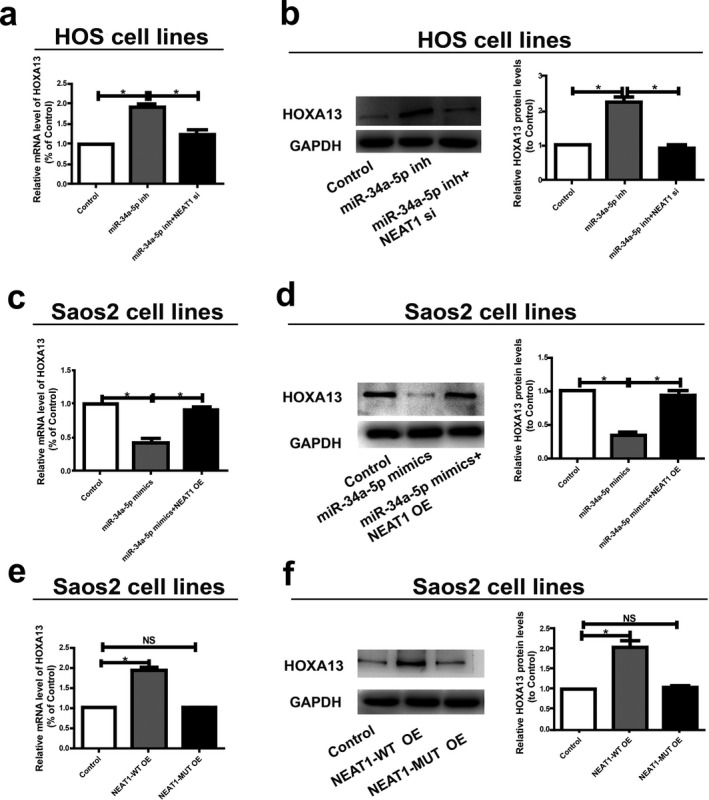
*NEAT1*/miR‐34a‐5p axis is critical for the expression of *HOXA13*. (a) MiR‐34a‐5p inhibitor with or without *NEAT1* siRNA is transfected into HOS cells and the mRNA level of *HOXA13* is evaluated by qRT‐PCR. (b) Western blot analysis of *HOXA13* protein level following treatment of HOS cells with miR‐34a‐5p inhibitor or *NEAT1* siRNA. *GAPDH* is used as control. (c) Saos2 cells are transfected with miR‐34a‐5p with or without *NEAT1* overexpress plasmid and qRT‐PCR is applied to detect the relative mRNA levels of *HOXA13* compared with controls. (d) Relative protein level of *HOXA13* when transfected with miR‐34a‐5p mimics and reversed by *NEAT1* expression plasmid. (e) Relative mRNA level of *HOXA13* when transfected with *NEAT1*‐WT overexpression plasmid or *NEAT1*‐MUT overexpression plasmid. (f) Relative protein level of *HOXA13* when transfected with *NEAT1*‐WT overexpression plasmid or *NEAT1*‐MUT overexpression plasmid. Data are presented as mean ± *SD*. *p < 0.05, ns, no significantly difference

To elucidate whether *NEAT1* regulated *HOXA13* expression via targeting miR‐34a‐5p, we detected expression levels of *HOXA13* in osteosarcoma cells after altering *NEAT1* or miR‐34a‐5p expressions. Transfection with miR‐34a‐5p inhibitor in HOS cells upregulated *HOXA13* expression, which was reversed by cotransfection with miR‐34a‐5p inhibitor and *NEAT1* siRNA (Figure 5a and b). Furthermore, transfection with miR‐34a‐5p mimics in Saos2 cells inhibited *HOXA13* expression, which was reversed by cotransfection with miR‐34a‐5p mimics and *NEAT1* overexpression plasmid (Figure 5c and d). Subsequently, Saos2 cells were transfected with *NEAT1* overexpression plasmid and its mutant overexpression plasmid, followed by the determination of *HOXA13* expression. Both qRT‐PCR and Western blot results showed that overexpression of wild‐type *NEAT1* upregulated *HOXA13* expression in osteosarcoma cells, whereas mutant‐type *NEAT1* did not disrupt base pairing between *NEAT1* and miR‐34a‐5p (Figure 5e and f). To sum up, our findings confirm that *NEAT1* positively regulates the expression of *HOXA13* by directly binding to miR‐34a‐5p.

### 3.6 *NEAT1*/miR‐34a‐5p axis regulates behaviors of osteosarcoma cells

**Figure 6:**
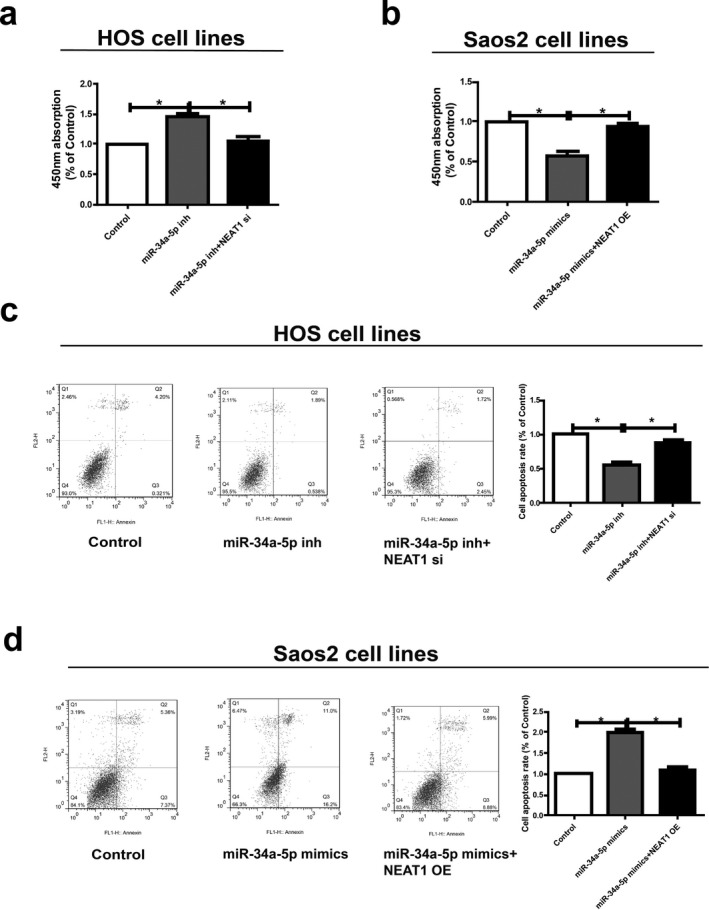
*NEAT1* regulates cell function through miR‐34a‐5p. (a and b) Determination of the proliferation of HOS and Saos2 cells via CCK‐8 assay. (c and d) The apoptosis ability with respect to changes of HOS and Saos2 cell lines after different transfection. Data are presented as mean ± *SD*. *p < 0.05

We next explored whether miR‐34a‐5p could affect proliferative and apoptosis potentials of HOS and Saos2 cells. Downregulation of miR‐34a‐5p in HOS cells markedly promoted proliferative and inhibited apoptosis potentials compared to controls, which were partially reversed by cotransfection with miR‐34a‐5p inhibitor and *NEAT1* siRNA (Figure 6a and c). In addition, overexpression of miR‐34a‐5p inhibited proliferative and promoted apoptosis potentials of Saos2 cells, and were partially reversed by *NEAT1* overexpression (Figure 6b and d). Based on the above results, *NEAT1*/miR‐34a‐5p axis exerts great effects on regulating behaviors of osteosarcoma cells.

## 4 DISCUSSION

Osteosarcoma is ranked among the leading causes of cancer‐related death in the pediatric age group. The cancer's low prevalence and its large tumor heterogeneity make it difficult to obtain meaningful progress in patient's survival (Botter, Neri, & Fuchs, 2014). LncRNA has been shown to be a regulator of various cellular processes. Dysregulated lncRNAs have been identified to be related to the disease development (Yu, Chuang, & Kuo, 2018). *NEAT1* has been confirmed to participate in cell proliferation (Wang, Wang, Zhang, Deng, & Long, 2017; Zhu et al., 2019), so we hypothesized that *NEAT1* may be involved in the pathogenesis of osteosarcoma.

Our study showed a higher expression of *NEAT1* in osteosarcoma cells relative to normal osteoblast cells. In addition, downregulation of *NEAT1* expression remarkably reduced proliferative and induced apoptosis capacities, suggesting that *NEAT1* was an important regulator in the growth of osteosarcoma cells as an oncogene. Therefore, explorations on the effect of *NEAT1* on accelerating growth of osteosarcoma cells are of great significance for in‐depth studies of the occurrence and development of osteosarcoma.

Through separation of cytoplasm and nucleus, we confirmed that *NEAT1* was mainly distributed in cell cytoplasm, indicating that *NEAT1* may serve as a ceRNA. Subsequently, RIP and dual‐luciferase reporter gene assay clarified that *NEAT1* could bind to miR‐34a‐5p. So far, miR‐34a‐5p has been proved to be lowly expressed in ovarian cancer and glioma (Ding, Wu, Tao, & Peng, 2017; Xu et al., 2018). Our study demonstrated that miR‐34a‐5p was downregulated in osteosarcoma cells, and transfection with miR‐34a‐5p mimics promoted apoptosis and suppressed proliferative capacities of osteosarcoma cells, which could be reversed by *NEAT1* overexpression. We believed that both *NEAT1* and miR‐34a‐5p may participate in the development and progression of osteosarcoma.

*HOX* genes play a fundamental role in the development of the vertebrate central nervous system, axial skeleton, limbs, gut, urogenital tract and external genitalia, but mutations in two of the 39 human *HOX* genes (*HOXD13* mutated in synpolydactyly, and *HOXA13* mutated in hand‐foot‐genital syndrome) have been shown to cause congenital malformations (Goodman & Scambler, 2001; Grier et al., 2005). Studies have shown that *HOXA13* exerts an important regulatory effect on organ development, cell differentiation, and tumorigenesis (Luo, Rhie, Lay, & Farnham, 2017). Quagliata et al. (2018) declared that high expression of *HOXA13* is correlated with poorly differentiated hepatocellular carcinomas and modulates sorafenib response in in vitro models. Hu, Chen, Cheng, Li, & Zhang (2017) suggested that dysregulated expression of homebox gene *HOXA13* is related to the poor prognosis in bladder cancer. Accordingly, our study verified that upregulated *NEAT1* increased expression of *HOXA13*, the target gene of miR‐34a‐5p, further leading to abnormal proliferation and apoptosis of osteosarcoma cells.

To sum up, *NEAT1* functioned as a competitive endogenous RNA to regulate *HOXA13* expression by sponging miR‐34a‐5p, thus regulating the development of osteosarcoma.

## CONFLICT OF INTEREST

None.
